# Pulp Capping Again

**Published:** 1878-02

**Authors:** W. C. Barrett

**Affiliations:** Buffalo. N. Y.


### ARTICLE IV.

### *Pulp Capping Again.*

BY DR. W. C. BARRETT, BUFFALO. N. Y.

My first task, when a person presents himself with an aching tooth, is to make a careful diagnosis. I enlarge the orifice of the cavity (we are pre-supposing a carious tooth,) remove all extraneous matter, and test the tooth for vitality, by injecting a stream of cold water. When satisfied that there is a living pulp, I thoroughly syringe out the cavity with tepid water, containing enough Carbonate of Soda to make it slightly alkaline. This will almost always relieve any acute pain. I then apply the rubber dam, and very carefully excavate, avoiding the removal of the cap of decalcified dentine, which perhaps covers the irritated pulp. After removing all decay, and if the pain has been but slight and recent, and there be a cover of but partially disintegrated dentine, the case is a simple one. I cover the decalcified dentine with a paste of the Lactophosphate of Lime, and upon this I place an Oxy-chloride of Zinc filling, and dismiss the patient for two or three months, at the end of which time I again apply the rubber-dam, remove the Oxy chloride of Zinc, and examine. If the softened dentine has become firm, and there is no tenderness to pressure, I cover with Oxy-chloride of Zinc, as a protection against thermal changes, and fill with gold. If, however, upon removing the temporary filling I find little or no improvement, but simply a state of quiescence, I again apply the Lacto-phosphate of Lime, and reinsert the temporary filling, dismissing the patient for another interval. This also will be nearly the course of treatment when a pulp is accidentally wounded by the instrument during excavation, though I usually interpose between this so wounded pulp and *Lacto Calcis Phos.*, some soothing emollient like Glycerine. In no such case would I further irritate the wounded pulp by cauterizing it with Creasote or Carbolic acid, whose influence could be only deleterious.

If upon my first examination, I find that the dentine immediately over the pulp is so far disintegrated as to be unfit to serve for a covering, or if there has been so much of pain as to indicate considerable or long continued inflammation, the case becomes more complicated. Removal of the thoroughly decayed dentine perhaps reveals actual exposure of pulp tissue. The first thing now is to make a more careful diagnosis, and see if there be any sloughing of the pulp. This is done by a minute examination under a magnifying glass, the cavity, of course, being kept entirely dry by means of the rubber-dam. If there be manifestly a loss of tissue, or an exhibition of pus, the superficial layer of odontoblast cells is clearly obliterated, and I can hope for no deposition of secondary dentine. A different method of treatment is in such cases demanded. If, however, the pulp chamber be normally filled with a pulp which appears vascular, and with a clearly defined margin, and not too sensitive to the touch, I commence treatment looking towards a return to health. In diagnosing the condition of the pulp when fairly exposed, I lay particular stress upon its sensibility. There is a normal and an abnormal sensitiveness. In a state of health the dental pulp has comparatively little feeling. The dentist, in such cases, is but seldom warned by the sensations of his patient of a near approach to that organ. He may quite deeply wound it with but little shrinking on his part. He may slightly puncture it without his knowledge. But when it is in an irritable and inflamed state, the patient convulsively starts at but the sweeping of a wave of air across it. A conception of the normal sensation resident in a healthy pulp is thus necessary to a clear diagnosis—a knowledge which, judging from the remarks common in the meetings of dental societies, but few dentists are possessed of. Satisfying myself from observation and trial of the dental pulp, that it is a case of simple exposure, unaccompained by sloughing, my first care is to see that every particle of debris or irritating foreign matter, is removed from its neighborhood. The next step is to attempt the reduction of the inflammation, and to forestall the effusion of serum or lymph. The borders of the opening into the pulp chamber are carefully trimmed, and the exposed surface is repeatedly washed with tepid water. It is then carefully dried, and bathed with dilute *Tinct. Aconite Rad.*, a thin coating of Glycerine or Collodion flowed over it, it is carefully sealed up with wax or a solution of Gutta Percha in Chloroform, and left for a few days quiet, directions being given to avoid the use of the tooth in mastication. If, on my next examination, the pulp is but normally sensitive, has ceased to be painful, and the irritated, inflamed condition has given way to a better state of affairs, preparatory treatment ceases; otherwise it is continued. When I judge it safe, I proceed to cap. This is a very simple operation, and consists in covering the exposed point with the Lacto-phosphate of Lime, over which is carefully flowed a thin solution of Gutta Percha dissolved in

Chloroform. The cavity is then filled with Oxy-chloride of Zinc, which is allowed to remain for three months. I then readjust the rubber-dam, and carefully remove a part of the Oxy-chloride of Zinc, so as to expose the periphery of the cavity. If that portion of the dentine which is over the cornua of the pulp be normally sensitive, it is sufficient indication that the desired result has been reached, and I fill with gold, leaving the main body of the Oxy-chloride of Zinc filling as a firm bridge over my capping, and my case is thus brought to a conclusion. I do not claim universal success by these means. I cannot raise the dead to life, nor can I save those pulps which are fatally stricken; but I think my practice is founded on reason, and it meets with reasonable success. When there is sloughing, or breaking down of tissue, different measures must be resorted to, which I have not now time to detail. Of course, this method of treatment presupposes free access to the cavity of decay, for the dentist who attempts the cure of any diseased tooth, without first securing proper access, and becoming master of the situation, is but a poor bungler, who deserves failure. I am careful not to irritate the pulp in any manner, for my hopes of final triumph rest upon the quiet soothing of the disturbed territory into a tranquil rest. It is, then, needless for me to say that all such empirical measures as puncturing the pulp to deplete it, find no favor with me. Any traumatic lesion can of course only complicate matters, as it produces a wound which must be healed, while the relief gained is but momentary.

The Lacto-phosphate of Lime I prepare myself, as wanted. The essentials are, a vial of Merck's Lactic Acid, another of the Magma Phosphate, which must be kept under water, and a little of the dry precipitate. On a piece of glass, place one drop of the Lactic acid; give it as much of the Magma as it will digest, and then reduce to the proper consistency by adding the precipitated Phosphate. I use this as the immediate cover to the exposed tissue, because it seems to be the most congenial, and least liable to quarrel with it. I shall be glad at some future time to relate to you my method of dealing with pulps when slight retrogradation has commenced, but I fear I have written all that you will at one time read with patience.—*Dental Advertiser.*

